# Sclera and Iris Color Interact to Influence Gaze Perception

**DOI:** 10.3389/fpsyg.2021.632616

**Published:** 2021-03-09

**Authors:** Jessica L. Yorzinski, Christopher A. Thorstenson, Trezze P. Nguyen

**Affiliations:** ^1^Department of Ecology and Conservation Biology, Texas A&M University, College Station, TX, United States; ^2^Department of Psychology and Wisconsin Institute for Discovery, University of Wisconsin-Madison, Madison, WI, United States; ^3^Department of Wildlife and Fisheries Sciences, Texas A&M University, College Station, TX, United States

**Keywords:** depigmented sclera, gaze enhancement hypothesis, gaze perception, human eye, iris color

## Abstract

The white sclera is important in facilitating gaze perception in humans. Iris color may likewise influence gaze perception but no previous studies have directly assessed its effect. We therefore examined how the interaction between sclera and iris color influences human gaze perception. We recorded the eye movements of human participants as they performed a visual search task with human faces exhibiting directed or averted gaze. The faces either exhibited light or dark irises. In addition, the faces had sclera that were depigmented (white) or pigmented (matched the color of the iris). We found that participants were quick and accurate in evaluating gaze regardless of iris color in faces with depigmented sclera. When the sclera were pigmented, participants were slower to evaluate the gaze of faces with both light and dark irises but these effects were most pronounced in the faces with dark irises. Furthermore, participants were generally less accurate in assessing faces with pigmented sclera when the irises were dark rather than light. Our results suggest that depigmented sclera are especially important for gaze perception in faces with dark irises. Because depigmented sclera likely evolved at a time when ancestral humans exhibited dark irises, the depigmented sclera may have been crucial for efficient and accurate gaze perception in ancestral humans.

## Introduction

The white sclera is a prominent feature of human eye morphology (Kobayashi and Kohshima, 2001). It can provide information about age, health status and attractiveness. People with whiter sclera are perceived as younger, healthier, and more attractive than people with yellower or redder sclera (Provine et al., 2011, 2013; Russell et al., 2014). White sclera can also facilitate gaze perception by enhancing the ability of people to evaluate where others are directing their overt attention (Kobayashi and Kohshima, 2001). Gaze perception is critical in many social contexts, allowing individuals to follow the gaze of others to ecologically-relevant stimuli (such as conspecifics, predators, or food) within the environment (Shepherd et al., 2006; Dalmaso et al., 2020). Gaze perception also allows individuals to assess whether others are looking at them directly, which can indicate intentions, foster social interaction, and guide social behavior (Emery, 2000; Senju and Hasegawa, 2005; Frischen et al., 2007).

The white sclera facilitates gaze perception in humans because the white sclera against the relatively darker irises induces visual contrast such that eye movements are highly salient (Ricciardelli et al., 2000; Kobayashi and Kohshima, 2001; Tomasello et al., 2007; Yorzinski and Miller, 2020). In particular, contrast polarity plays an important role in gaze perception (Ricciardelli et al., 2000; Sinha, 2000; Tipples, 2005; Olk et al., 2008). Humans are highly accurate in evaluating gaze when contrast polarity is normal (i.e., dark pupils/irises and light sclera) but relatively inaccurate when contrast polarity is reversed (i.e., light pupils/irises and dark sclera). These results suggest that gaze perception is largely determined by where the darkest area of the eye is directed (but see Olk et al., 2008). Additional features of the eye regions, such as sclera luminance and size, can also impact gaze perception. When the luminance of one side of the sclera is darkened, humans often perceive that face to be looking toward the side with the darkened sclera even though the pupils are directed straight ahead (Ando, 2002); this effect is lessened when the luminance of the skin surrounding the darkened sclera is also darkened (Ando, 2004). Humans also orient better to gaze when eyelids are raised to expose more of the sclera (Tipples, 2005). In contrast, the sclera in some non-human primates is pigmented or less exposed, and its role in gaze perception may therefore be minimized in these species (Kobayashi and Kohshima, 2001). For the same reasons that white sclera facilitate gaze perception in humans, iris color (and its interaction with sclera color) could potentially influence gaze perception (Kobayashi and Kohshima, 2001; Ueda et al., 2014).

Iris color in humans ranges from light blue to dark brown or black (Sturm and Frudakis, 2004). Light irises have only recently evolved, with the mutation responsible for light irises emerging ≈6,000–10,000 years ago (Eiberg et al., 2008). Regardless of sclera color, the gaze of individuals with light irises may be particularly easy to discern because light irises exhibit high contrast with black pupils. Conversely, the gaze of individuals with dark irises might be difficult to discern without a white sclera because dark irises exhibit low contrast with black pupils. It is unknown whether these contrast differences are important for gaze perception. It is possible that the white sclera is important for gaze perception in people with dark but not light irises. Given that scleral depigmentation likely occurred long before light irises evolved, the white sclera may have played an even greater role in ancestral human's gaze perception than previously considered. We are unaware of any studies that have examined the influence of both sclera and iris color on gaze perception.

We therefore examined how the interaction between sclera and iris color influences human gaze perception. We recorded the eye movements of human (*Homo sapiens*) participants as they searched for faces with directed gaze within arrays of faces with averted gaze or searched for faces with averted gaze within arrays of faces with directed gaze (using similar methodology as Yorzinski and Miller, 2020). The faces exhibited either light or dark irises. In addition, the faces had sclera that were depigmented (white) or pigmented (matched the iris color). The faces with pigmented sclera represent a phenotype that is similar to many primate species in which the sclera has a similar color as the iris color or the sclera is minimally exposed (Kobayashi and Kohshima, 2001). However, there are some primate species with more conspicuous sclera (Mayhew and Gómez, 2015; Perea-García et al., 2019). Our previous results found that sclera color influences gaze perception (Yorzinski and Miller, 2020) but the current study expands upon those results by examining the impact of both sclera color and iris color on gaze perception. Because contrast between the sclera, iris, and pupil all potentially contribute to gaze perception, it is important to consider each of their effects.

We presented the participants with faces that were large and upright, small and upright, or large and inverted. The large and upright faces simulated viewing faces in close interactions while the small and upright faces simulated viewing faces in distant interactions. Gaze perception is likely important at a range of distances, with close-up interactions being especially important for monitoring social relationships (Anderson et al., 2005; Zuberbühler, 2008) and more distant interactions being critical for predator detection and hunting (Ueda et al., 2014). The inverted faces preserved low-level visual properties but disrupted facial configuration (McKone et al., 2013; Yokoyama et al., 2014). Previous work has found that gaze perception is processed with component-based information from the eye region rather than configural processing of the entire face (Schwaninger et al., 2005). As such, gaze perception is often intact regardless of whether faces are upright or inverted (Myowa-Yamakoshi and Tomonaga, 2001; Tipples, 2005; Tomonaga and Imura, 2010).

When the faces exhibited depigmented sclera, we predicted that participants would be fast and accurate in evaluating gaze regardless of iris color because the contrast between the pupil and white sclera is high. When the faces exhibited pigmented sclera, we predicted that participants would be faster and more accurate in evaluating gaze in faces with light irises vs. dark irises because the contrast between the pupil and light irises/sclera is greater than the contrast between the pupil and dark irises/sclera. We expected that participants would be faster at evaluating gaze for the larger compared to smaller faces because gaze is more difficult to assess at greater distances (Martin and Jones, 1982). We also expected the general patterns to be upheld when the faces were inverted because gaze perception is often processed based on component information rather than configural information (Schwaninger et al., 2005).

## Materials and Methods

### Participants

Fifty-one participants (30 women and 21 men) enrolled in this study at Texas A&M University from September 2019 through January 2020. They were of Caucasian ethnicity and between the ages of 18 and 30 years old. We recruited the participants through emails. The participants were told that they would be participating in a study that explored gaze detection and they each earned $20. We tested the acuity (near vision card; Bernell, Inc.) and color vision (Good-lite color vision screening plates; plates 1–8) of the participants and they all had normal or corrected-to-normal acuity as well as normal color vision. The institutional review board of Texas A&M University (2019-0762D) approved this study and written consent was obtained from all participants.

### Equipment

The eye movements of participants were recorded with a Tobii Pro screen-based eye-tracker (X2-60; Tobii Technology, Inc., Danderyd, Sweden; accuracy: 0.4°; data rate: 60 Hz; binocular tracking) that was run by a laptop computer (Dell Mobile Precision 7510). The stimuli were displayed on a self-calibrating LCD monitor (Eizo, ColorEdge CG2730; 2,560 × 1,440 pixels; luminance: 120 cd m^−2^; gamma = 2.2; white point = 6,500 K) using custom eye-tracking software (Tobii Studio 3.4, Tobii Technology). The eye-tracker non-invasively monitored the eye movements of participants and recorded the location of where they were looking. The participants were positioned ≈60 cm from the screen and rested their chins in a chin cup (UHCOTech HeadSpot) to minimize head movements. Participants were told that we were measuring the size of their pupils but were not told that their eye movements were being monitored until after they completed the trial. The eye-tracker was calibrated (five points) before each trial began. We used the Tobii Velocity-Threshold Identification filter (I-VT filter; gap fill-in: 75 ms; eye selection: average; velocity calculator window: 20 ms; I-VT classifier threshold: 30°/s; merge adjacent time: 75 ms; merge adjacent angle: 0.5°) to classify fixations and saccades. This filter classifies eye movements as fixations or saccades based upon the velocity of eye movements. Eye movements below and above the velocity threshold (30°/s, in this study) are classified as fixations and saccades, respectively. Eye-tracking data consisted of coordinates of where participants were known to be looking during each sampling point.

### Experimental Stimuli

The experimental stimuli consisted of photographs of human faces (Oslo Face Database; Chelnokova et al., 2014). The people in the photographs exhibited neutral expressions with their heads facing directly toward the camera and their eyes either directed or averted. Because the stimuli were taken from a face database, the people in the stimuli were likely unfamiliar to the participants.

**Figure 1:**
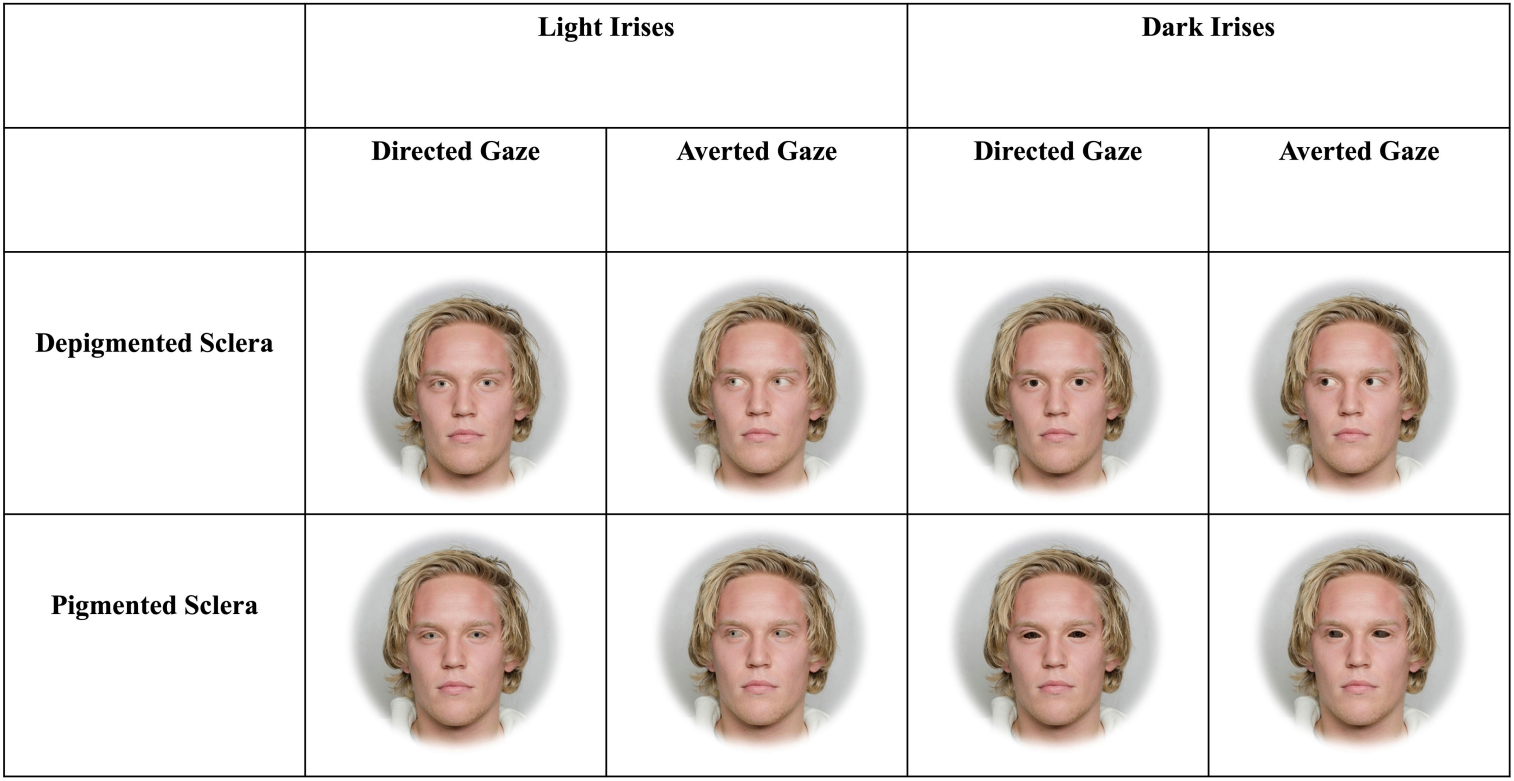
Example stimuli depicting faces with light and dark irises as well as sclera that are depigmented or pigmented. The man in the stimuli below gave informed consent for his photograph to be used for scientific purposes (Chelnokova et al., 2014).

**Figure 2:**
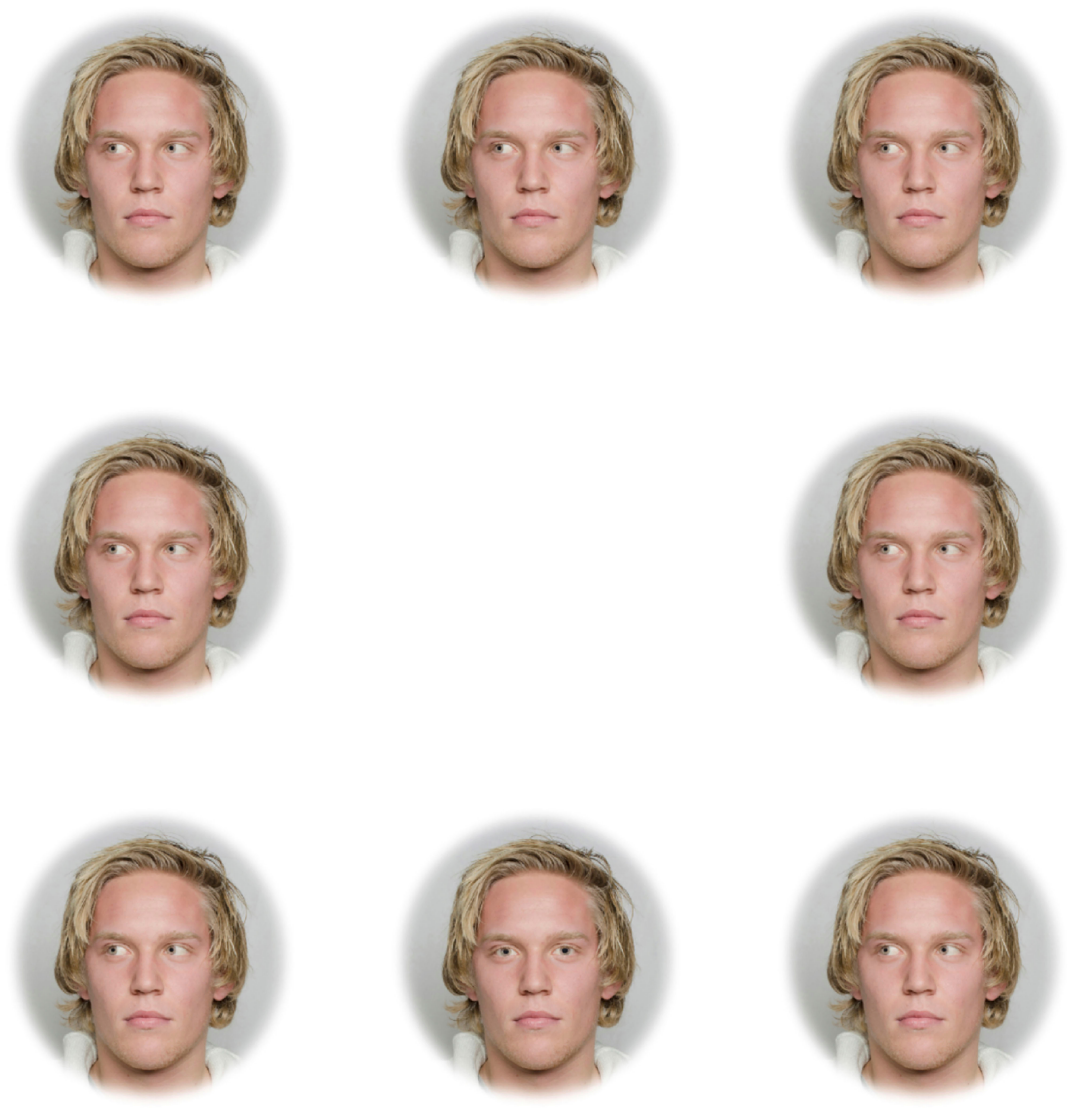
Example of array used in the search task for the Directed Depigmented set with light irises.

We created three blocks of faces each with four sets for two treatments (Figures 1, 2). The two treatments consisted of faces that exhibited dark or light irises. Matlab was used to manipulate the color of the irises. The lightness (CIELAB L^*^) of the original iris image was increased or decreased such that the light irises had an average L^*^ of ≈57, while the dark irises had an average L^*^ of ≈22. These values correspond to the lightest and darkest original iris colors from the stimuli we used, respectively. This was done so that the heterogeneity of iris color (i.e., small variations in color across the iris), texture, and specular highlights were preserved. This also ensured that the lightness values of the manipulated irises (i.e., all dark or light irises) were equivalent regardless of the lightness of the original target's iris. Within targets, the chromaticity (CIELAB a^*^ and b^*^) of iris color between dark and light irises were equal. Photoshop was used to apply these color changes to the original image, such that the color changes were only applied to the iris. The CIELAB color space is modeled on the human visual system and designed to be perceptually uniform (Fairchild, 2013).

The three blocks included faces that were upright and subtended 5.7° (“large and upright”), faces that were upright and subtended 2.85° (“small and upright”), and faces that were inverted and subtended 5.7° (“large and inverted”). Within each of these three blocks, four sets of 8-array photographs were shown: (1) face with a directed gaze and depigmented sclera within an array of faces with averted gaze and depigmented sclera (“Directed Depigmented”), (2) face with a directed gaze and pigmented sclera within an array of faces with averted gaze and pigmented sclera (“Directed Pigmented”), (3) face with an averted gaze and depigmented sclera within an array of faces with directed gaze and depigmented sclera (“Averted Depigmented”), and (4) face with an averted gaze and pigmented sclera within an array of faces with directed gaze and pigmented sclera (“Averted Pigmented;” Figures 1, 2). In the faces with pigmented sclera, the mean color of the sclera matched the mean color of the iris. The sclera color was manipulated in the same way as was iris color, in order to preserve the heterogeneity of sclera color, texture, and specular highlights. Within each set, 40 different faces were shown. Overall, a given participant viewed 960 arrays (2 treatments × 3 blocks × 4 sets × 40 images per set).

### Experimental Procedure

The experimenter first asked participants to perform a practice trial so they could become familiar with the procedure. In the practice trial, the participants were shown arrays of photographs of domestic cats (*Felis catus*). In each array, seven of the cat faces had eyes that were directed and one had eyes that were averted. Participants were instructed to search for the cat face that had its eyes averted as quickly and accurately as possible and then indicate their response using a custom keypad (model: CP24-USBHID; Genovation, Irvine, CA). The keypad had eight keys that were arranged in the same configuration as the experimental stimuli (a 3 × 3 grid with no key in the middle). They were also shown arrays of photographs of cats with the opposite arrangement: seven of the cat faces had eyes that were averted and one had eyes that were directed. They were instructed to search for the cat face that had its eyes directed as quickly and accurately as possible and then indicate their response using the custom keypad. After they pressed the keypad, the next array appeared.

After completing the practice trial, participants performed the experimental task. The participants were instructed to search for the directed gaze within the arrays of one face with a directed gaze and seven faces with averted gaze (Directed Depigmented and Directed Pigmented) and then indicate their response using the custom keypad; or they were instructed to search for the averted gaze within the arrays of one face with an averted gaze and seven faces with directed gaze (Averted Depigmented and Averted Pigmented) and then indicate their response using the custom keypad. After they pressed the keypad, the next array appeared. A given subject completed both treatments (light and dark irises; treatment order randomized across participants). The blocks and sets within the treatments were randomized across participants.

### Measurements and Statistical Analysis

Using a customized MATLAB program, we drew regions of interest (ROIs) around each face. For each fixation coordinate, we determined which ROI it fell within to determine whether the participant was looking at the target face, distractor faces, or neither the target nor distractor faces. We calculated the amount of time that elapsed before participants fixated on the target (Latency to Fixate Target) and the amount of time that elapsed between the participants fixating the correct target and manually responding by pressing a key to indicate they detected the correct target (Latency to Press Key After Fixate Target). For each participant, we calculated the mean value of the metrics within each of the four sets (Directed Depigmented, Directed Pigmented, Averted Depigmented, and Averted Pigmented) of the three blocks (large, small, and inverted) for each treatment (light irises and dark irises). In arrays where the data indicated a subject never fixated the target, it was not possible to determine whether the participants did not fixate the target (and therefore did not correctly perform the task) or whether the eye-tracker failed to record the participants' gaze when they were fixating the target. We therefore excluded a given matrix from the analysis if a participant's fixations never fell within the target or if more than 10% of the gaze data was missing (9.1% matrices were excluded).

We analyzed our data using linear mixed-effects models with repeated measures in SAS (PROC MIXED; Version 9.4; SAS Institute Inc., Cary, NC). The dependent variable was the latency to fixate the target face. The independent variables were the block (“large and upright,” “small and upright,” and “large and inverted”), set (Directed Depigmented, Directed Pigmented, Averted Depigmented, and Averted Pigmented), treatment (light and dark iris) and their interaction. Block order (order in which the blocks were presented for each participant), age, and gender of the participants were covariates. Participant identity was included within the models to account for repeated measures. We performed *a priori* contrasts to compare the latency to detect the target face relative to whether the iris was light or dark as well as between the depigmented and pigmented sclera; we performed 36 comparisons and used the false discovery rate correction to evaluate statistical significance (Benjamini and Hochberg, 1995). We performed similar repeated-measures mixed linear models using the latency to indicate their response (either correct or incorrect; via a key press) after fixating the target as the dependent variable. In addition, we performed a similar generalized linear mixed model (PROC GLIMMIX; Poisson distribution) using the percentage of correct responses as the dependent variable.

## Results

**Figure 3:**
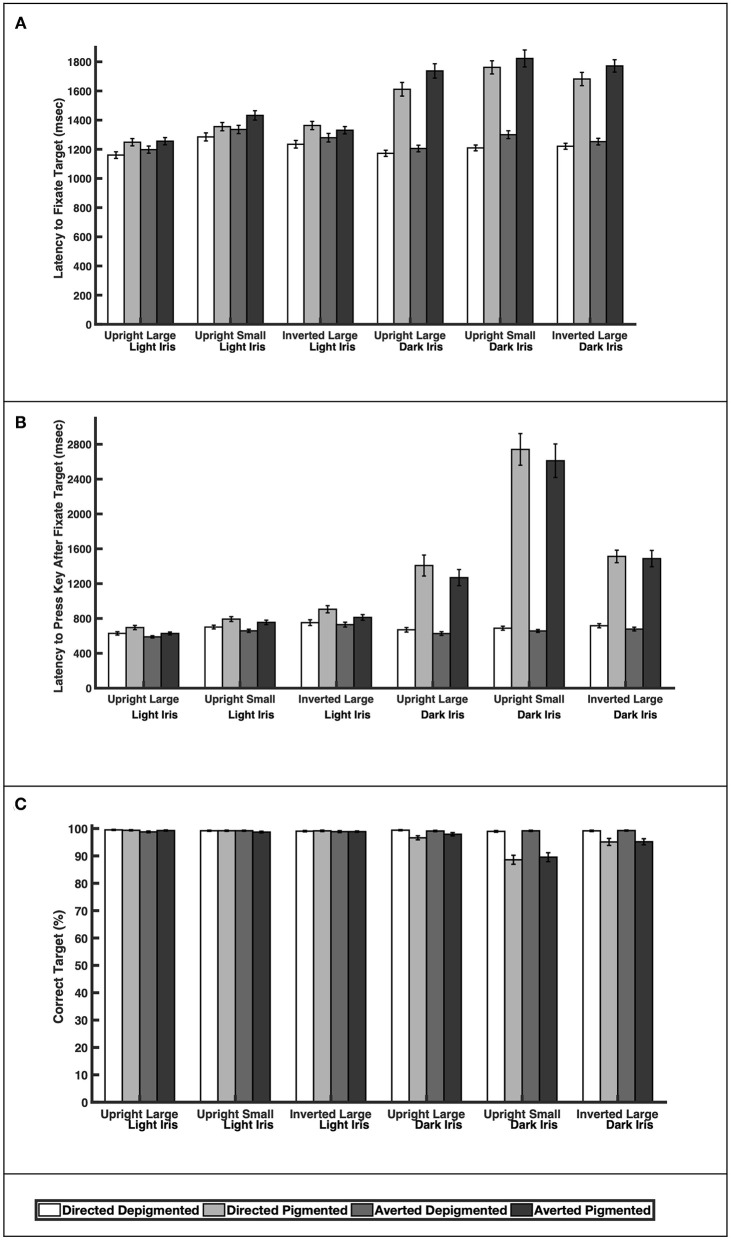
The **(A)** latency to initially fixate the target, **(B)** latency to indicate choice (via key press) after initially fixating the target, and **(C)** percentage of correct responses for large and upright faces, small and upright faces, and large and inverted faces. Means and standard errors are shown.

When the faces exhibited depigmented sclera, participants were quick to fixate the target faces regardless of iris lightness (Figure 3A; Table 1; Latency to Fixate Target). They fixated the target faces with light or dark irises similarly for large faces [directed: *F*_(1, 39)_ = 0.64, *p* = 0.52; averted: *F*_(1, 39)_ = 0.35, *p* = 0.73], small faces [averted: *F*_(1, 39)_ = 1.31, *p* = 0.20], and inverted faces [directed: *F*_(1, 39)_ = 0.57, *p* = 0.57; averted: *F*_(1, 39)_ = 1.05, *p* = 0.30] but were slightly faster to fixate the small, directed faces with dark irises compared to the small, directed faces with light irises [*F*_(1, 39)_ = 2.67, *p* = 0.011]. When the faces exhibited pigmented sclera, participants were quicker to fixate the target faces when the irises were light rather than dark for large faces [directed: *F*_(1, 39)_ = 8.92, *p* < 0.0001; averted: *F*_(1, 39)_ = 10.31, *p* < 0.0001], small faces [directed: *F*_(1, 39)_ = 9.91, *p* < 0.0001; averted: *F*_(1, 39)_ = 8.19, *p* < 0.0001], and inverted faces [directed: *F*_(1, 39)_ = 7.20, *p* < 0.0001; averted: *F*_(1, 39)_ = 10.75, *p* < 0.0001]. Overall, participants were faster to fixate the target faces when the sclera were depigmented compared to pigmented (*p* < 0.03). These same patterns were upheld in regards to participants' latency to manually indicate a response after fixating the target except that they indicated their responses at similar speeds for the small, directed faces with light and dark irises when the sclera were depigmented [*F*_(1, 39)_ = 0.57, *p* = 0.57; Figure 3B; Table 1; Latency to Press Key After Fixate Target].

**Table 1:** The effect of block, set, treatment, block order, age, and gender on the latency to fixate the target and latency to press a key after fixating the target.

	**Numerator *df*, denominator *df***	**Latency to fixate target**	**Latency to press key after fixate target**
**OVERALL MODEL**			
Block	2, 39	35.29 (<0.0001)^*^	44.1 (<0.0001)^*^
Set	3, 39	121.13 (<0.0001)^*^	74.14 (<0.0001)^*^
Treatment	1, 39	107.09 (<0.0001)^*^	117.04 (<0.0001)^*^
Block^*^Set^*^Treatment	17, 39	19.16 (<0.0001)^*^	22.71 (<0.0001)^*^
Block order	9, 39	2.67 (0.016)^*^	12.28 (<0.0001)^*^
Age	1, 39	0.04 (0.83)	4.24 (0.046)^*^
Gender	1, 39	2.61 (0.11)	0.22 (0.64)
**COMPARISONS**			
**Large and Upright**			
Directed Depigmented Light Iris vs. Directed Depigmented Dark Iris	1, 39	0.64 (0.52)	1.42 (0.16)
Directed Pigmented Light Iris vs. Directed Pigmented Dark Iris	1, 39	8.92 (<0.0001)^*^	6.17 (<0.0001)^*^
Averted Depigmented Light Iris vs. Averted Depigmented Dark Iris	1, 39	0.35 (0.73)	1.96 (0.058)
Averted Pigmented Light Iris vs. Averted Pigmented Dark Iris	1, 39	10.31 (<0.0001)^*^	6.86 (<0.0001)^*^
Directed Depigmented Dark Iris vs. Directed Pigmented Dark Iris	1, 39	10.14 (<0.0001)^*^	6.86 (<0.0001)^*^
Averted Depigmented Dark Iris vs. Averted Pigmented Dark Iris	1, 39	11.96 (<0.0001)^*^	7.26 (<0.0001)^*^
Directed Depigmented Light Iris vs. Directed Pigmented Light Iris	1, 39	4.95 (<0.0001)^*^	4.72 (<0.0001)^*^
Averted Depigmented Light Iris vs. Averted Pigmented Light Iris	1, 39	2.63 (0.012)^*^	3.51 (0.0012)^*^
Directed Depigmented Light Iris vs. Averted Depigmented Light Iris	1, 39	2.05 (0.047)	2.89 (0.0062)^*^
Directed Pigmented Light Iris vs. Averted Pigmented Light Iris	1, 39	0.34 (0.74)	3.65 (0.0008)^*^
Directed Depigmented Dark Iris vs. Averted Depigmented Dark Iris	1, 39	1.58 (0.12)	2.68 (0.011)^*^
Directed Pigmented Dark Iris vs. Averted Pigmented Dark Iris	1, 39	3.41 (0.0015)^*^	2.08 (0.044)
**Small and Upright**			
Directed Depigmented Light Iris vs. Directed Depigmented Dark Iris	1, 39	2.67 (0.011)^*^	0.57 (0.57)
Directed Pigmented Light Iris vs. Directed Pigmented Dark Iris	1, 39	9.91 (<0.0001)^*^	10.97 (<0.0001)^*^
Averted Depigmented Light Iris vs. Averted Depigmented Dark Iris	1, 39	1.31 (0.20)	0.07 (0.94)
Averted Pigmented Light Iris vs. Averted Pigmented Dark Iris	1, 39	8.19 (<0.0001)^*^	9.85 (<0.0001)^*^
Directed Depigmented Dark Iris vs. Directed Pigmented Dark Iris	1, 39	14.64 (<0.0001)^*^	12.09 (<0.0001)^*^
Averted Depigmented Dark Iris vs. Averted Pigmented Dark Iris	1, 39	9.71 (<0.0001)^*^	10.46 (<0.0001)^*^
Directed Depigmented Light Iris vs. Directed Pigmented Light Iris	1, 39	2.77 (0.0085)^*^	5.11 (<0.0001)^*^
Averted Depigmented Light Iris vs. Averted Pigmented Light Iris	1, 39	3.99 (0.0003)^*^	4.27 (0.0001)^*^
Directed Depigmented Light Iris vs. Averted Depigmented Light Iris	1, 39	2.42 (0.02)^*^	2.49 (0.017)^*^
Directed Pigmented Light Iris vs. Averted Pigmented Light Iris	1, 39	3.00 (0.0047)^*^	1.37 (0.18)
Directed Depigmented Dark Iris vs. Averted Depigmented Dark Iris	1, 39	3.93 (0.0003)^*^	1.88 (0.07)
Directed Pigmented Dark Iris vs. Averted Pigmented Dark Iris	1, 39	1.48 (0.15)	1.02 (0.31)
**Large and Inverted**			
Directed Depigmented Light Iris vs. Directed Depigmented Dark Iris	1, 39	0.57 (0.57)	1.03 (0.31)
Directed Pigmented Light Iris vs. Directed Pigmented Dark Iris	1, 39	7.20 (<0.0001)^*^	8.06 (<0.0001)^*^
Averted Depigmented Light Iris vs. Averted Depigmented Dark Iris	1, 39	1.05 (0.30)	1.66 (0.11)
Averted Pigmented Light Iris vs. Averted Pigmented Dark Iris	1, 39	10.75 (<0.0001)^*^	6.75 (<0.0001)^*^
Directed Depigmented Dark Iris vs. Directed Pigmented Dark Iris	1, 39	12.29 (<0.0001)^*^	12.14 (<0.0001)^*^
Averted Depigmented Dark Iris vs. Averted Pigmented Dark Iris	1, 39	14.75 (<0.0001)^*^	8.89 (<0.0001)^*^
Directed Depigmented Light Iris vs. Directed Pigmented Light Iris	1, 39	4.98 (<0.0001)^*^	5.23 (<0.0001)^*^
Averted Depigmented Light Iris vs. Averted Pigmented Light Iris	1, 39	2.28 (0.029)^*^	3.75 (0.0006)^*^
Directed Depigmented Light Iris vs. Averted Depigmented Light Iris	1, 39	1.75 (0.088)	1.09 (0.28)
Directed Pigmented Light Iris vs. Averted Pigmented Light Iris	1, 39	1.46 (0.15)	3.67 (0.0007)^*^
Directed Depigmented Dark Iris vs. Averted Depigmented Dark Iris	1, 39	1.62 (0.11)	2.25 (0.030)^*^
Directed Pigmented Dark Iris vs. Averted Pigmented Dark Iris	1, 39	2.62 (0.013)^*^	0.26 (0.79)

*F-values are displayed for the overall model and t-values are displayed for the comparisons; p-values are indicated in parentheses and statistically significant comparisons are indicated with an asterisk*.

Participants were highly accurate in gaze perception (Figure 3C; Table 2). When the faces exhibited depigmented sclera, participants averaged over 98% accuracy in identifying the correct face target. Regardless of iris lightness, they were equally accurate when evaluating large faces [directed: *F*_(1, 850)_ = 0.12, *p* = 0.91; averted: *F*_(1, 850)_ = 0.34, *p* = 0.73], small faces [directed: *F*_(1, 850)_ = 0.25, *p* = 0.81; averted: *F*_(1, 850)_ = 0.04, *p* = 0.97], and inverted faces [directed: *F*_(1, 850)_ = 0.13, *p* = 0.90; averted: *F*_(1, 850)_ = 0.43, *p* = 0.67]. When the faces exhibited pigmented sclera, participants were more accurate in assessing faces with light irises compared to dark irises when the faces were large [directed: *F*_(1, 850)_ = 2.93, *p* = 0.0035], small [directed: *F*_(1, 850)_ = 11.57, *p* < 0.0001; averted: *F*_(1, 850)_ =9.98, *p* < 0.0001], and inverted [directed: *F*_(1, 850)_ =4.34, *p* < 0.0001; averted: *F*_(1, 850)_ = 3.97, *p* < 0.0001] except they were equally accurate in evaluating large, averted faces with light and dark irises [*F*_(1, 850)_ = 1.44, *p* = 0.15]. When the irises were light, participants were similarly accurate when the sclera were depigmented or pigmented (*p* > 0.60); when the irises were dark, they were more accurate (except for large, averted faces) when the sclera were depigmented compared to pigmented (*p* < 0.003).

**Table 2:** The effect of block, set, treatment, block order, age, and gender on the percentage of correct responses.

	**Numerator *df*, denominator *df***	**Correct response**
**OVERALL MODEL**		
Block	2, 100	24.90 (<0.0001)^*^
Set	3, 150	34.68 (<0.0001)^*^
Treatment	1, 50	97.53 (<0.0001)^*^
Block^*^Set^*^Treatment	17, 850	13.61 (<0.0001)^*^
Block Order	9, 39	4.86 (0.0002)^*^
Age	1, 39	1.12 (0.30)
Gender	1, 39	7.4 (0.0097)^*^
**Comparisons**		
**Large and Upright**		
Directed Depigmented Light Iris vs. Directed Depigmented Dark Iris	1, 850	0.12 (0.91)
Directed Pigmented Light Iris vs. Directed Pigmented Dark Iris	1, 850	2.93 (0.0035)^*^
Averted Depigmented Light Iris vs. Averted Depigmented Dark Iris	1, 850	0.34 (0.73)
Averted Pigmented Light Iris vs. Averted Pigmented Dark Iris	1, 850	1.44 (0.15)
Directed Depigmented Dark Iris vs. Directed Pigmented Dark Iris	1, 850	2.97 (0.0031)^*^
Averted Depigmented Dark Iris vs. Averted Pigmented Dark Iris	1, 850	1.27 (0.21)
Directed Depigmented Light Iris vs. Directed Pigmented Light Iris	1, 850	0.15 (0.88)
Averted Depigmented Light Iris vs. Averted Pigmented Light Iris	1, 850	0.52 (0.60)
Directed Depigmented Light Iris vs. Averted Depigmented Light Iris	1, 850	0.77 (0.44)
Directed Pigmented Light Iris vs. Averted Pigmented Light Iris	1, 850	0.10 (0.92)
Directed Depigmented Dark Iris vs. Averted Depigmented Dark Iris	1, 850	0.31 (0.75)
Directed Pigmented Dark Iris vs. Averted Pigmented Dark Iris	1, 850	1.39 (0.17)
**Small and Upright**		
Directed Depigmented Light Iris vs. Directed Depigmented Dark Iris	1, 850	0.25 (0.81)
Directed Pigmented Light Iris vs. Directed Pigmented Dark Iris	1, 850	11.57 (<0.0001)^*^
Averted Depigmented Light Iris vs. Averted Depigmented Dark Iris	1, 850	0.04 (0.97)
Averted Pigmented Light Iris vs. Averted Pigmented Dark Iris	1, 850	9.98 (<0.0001)^*^
Directed Depigmented Dark Iris vs. Directed Pigmented Dark Iris	1, 850	11.32 (<0.0001)^*^
Averted Depigmented Dark Iris vs. Averted Pigmented Dark Iris	1, 850	10.47 (<0.0001)^*^
Directed Depigmented Light Iris vs. Directed Pigmented Light Iris	1, 850	0.01 (0.99)
Averted Depigmented Light Iris vs. Averted Pigmented Light Iris	1, 850	0.53 (0.60)
Directed Depigmented Light Iris vs. Averted Depigmented Light Iris	1, 850	0.01 (0.99)
Directed Pigmented Light Iris vs. Averted Pigmented Light Iris	1, 850	0.53 (0.60)
Directed Depigmented Dark Iris vs. Averted Depigmented Dark Iris	1, 850	0.22 (0.83)
Directed Pigmented Dark Iris vs. Averted Pigmented Dark Iris	1, 850	1.07 (0.28)
**Large and Inverted**		
Directed Depigmented Light Iris vs. Directed Depigmented Dark Iris	1, 850	0.13 (0.90)
Directed Pigmented Light Iris vs. Directed Pigmented Dark Iris	1, 850	4.34 (<0.0001)^*^
Averted Depigmented Light Iris vs. Averted Depigmented Dark Iris	1, 850	0.43 (0.67)
Averted Pigmented Light Iris vs. Averted Pigmented Dark Iris	1, 850	3.97 (<0.0001)^*^
Directed Depigmented Dark Iris vs. Directed Pigmented Dark Iris	1, 850	4.35 (<0.0001)^*^
Averted Depigmented Dark Iris vs. Averted Pigmented Dark Iris	1, 850	4.38 (<0.0001)^*^
Directed Depigmented Light Iris vs. Directed Pigmented Light Iris	1, 850	0.12 (0.90)
Averted Depigmented Light Iris vs. Averted Pigmented Light Iris	1, 850	0.01 (0.99)
Directed Depigmented Light Iris vs. Averted Depigmented Light Iris	1, 850	0.19 (0.85)
Directed Pigmented Light Iris vs. Averted Pigmented Light Iris	1, 850	0.30 (0.76)
Directed Depigmented Dark Iris vs. Averted Depigmented Dark Iris	1, 850	0.11 (0.91)
Directed Pigmented Dark Iris vs. Averted Pigmented Dark Iris	1, 850	0.08 (0.94)

*F-values are displayed for the overall model and t-values are displayed for the comparisons; p-values are indicated in parentheses and statistically significant comparisons are indicated with an asterisk*.

## Discussion

This study found that human participants were quick and accurate in evaluating gaze regardless of iris color in faces with depigmented sclera. When the faces exhibited pigmented sclera (sclera color matched the iris color), participants were slower to evaluate gaze and these effects were most pronounced in the faces with dark irises. Furthermore, participants were generally less accurate in assessing gaze in faces with pigmented sclera when the irises were dark rather than light. The overall effects were similar irrespective of whether the faces were upright or inverted, which is consistent with previous work suggesting that gaze perception involves component-based processing of the eye region rather than configural processing of the entire face (von Grünau and Anston, 1995; Myowa-Yamakoshi and Tomonaga, 2001; Tomonaga and Imura, 2010).

The depigmented sclera is particularly important for gaze perception in faces with dark irises. Participants could easily assess gaze direction when faces with dark irises exhibited depigmented sclera, regardless of whether the faces were large or small. In contrast, participants had difficulty in assessing gaze direction when the sclera were pigmented (sclera color matched the dark iris color). In fact, participants were 1.4× slower to fixate the target faces with dark irises and dark sclera compared to faces with dark irises and white sclera. They were also generally less accurate in assessing gaze direction in faces with pigmented sclera. When the faces were small, they averaged ≈90% accuracy in evaluating faces with dark irises and dark sclera (compared to 99% accuracy for faces with dark irises and white sclera). Because contrast between the pupil and dark iris is low, we predicted that gaze perception would be difficult when the sclera color matched the dark iris color and our findings supported this prediction. Similarly, people exhibit slow search times when the contrast of target symbols is low (Näsänen et al., 2001).

The depigmented sclera was also important for gaze perception in faces with light irises but the effect was significantly less pronounced. Because contrast between the pupil and light iris is relatively high, we predicted that gaze perception would remain accurate even when the sclera was pigmented (sclera color matched the light iris color). As expected, the participants were accurate in evaluating gaze direction in faces with light irises regardless of sclera pigmentation (over 99% accuracy). Participants were, however, slightly slower to find target faces with light irises and light sclera compared to faces with light irises and white sclera. Overall, gaze perception in faces with light irises was slightly faster when the sclera was depigmented but gaze was still accurately perceived with pigmented sclera.

We did not find strong support for the “stare-in-the-crowd” effect (von Grünau and Anston, 1995; Böckler et al., 2014), which posits that a face with directed gaze is easier to detect among faces with averted gaze compared with the opposite (i.e., a face with averted gaze among faces with directed gaze). When the faces exhibited depigmented sclera, participants were equally quick to fixate the target faces regardless of whether those faces were directed or averted except that they were faster to find the directed faces when the faces were small. Furthermore, participants were often slower to manually indicate their responses after fixating target faces with directed gaze vs. target faces with averted gaze. Other studies have also failed to find evidence supporting the “stare-in-the-crowd” effect (Cooper et al., 2013; Riechelmann et al., 2020).

Our results are consistent with previous studies demonstrating that eye morphology influences gaze perception (Langton et al., 2000; Ricciardelli et al., 2000; Ando, 2002, 2004; Jenkins, 2007; Olk et al., 2008; Anderson et al., 2016; Yorzinski and Miller, 2020). Humans are highly accurate in evaluating gaze when the pupil is darker than the sclera but they perform poorly when the contrast polarity is reversed (Ricciardelli et al., 2000; Sinha, 2000; Tipples, 2005; Olk et al., 2008). Similarly, we found that accuracy in gaze perception is generally high and is influenced by contrast in eye morphology. Especially for faces with dark irises, the depigmented sclera is critical for fast and accurate gaze assessment. Because depigmented sclera likely evolved at a time when ancestral humans exhibited dark irises (Eiberg et al., 2008), the depigmented sclera would probably have been particularly important for efficient and accurate gaze perception in these ancestral humans. Some non-human primates exhibit relatively dark irises with minimally exposed sclera (Kobayashi and Kohshima, 2001; but see Mayhew and Gómez, 2015; Perea-García et al., 2019), suggesting that gaze perception may be difficult for these species. Other non-human primates, however, exhibit light irises with minimally exposed sclera (Meyer et al., 2013) and may therefore be able to easily discriminate gaze. Future experiments could therefore examine how contrast in eye morphology influences gaze perception in non-human primates.

## Data Availability Statement

The datasets presented in this study can be found in online repositories. The names of the repository/repositories and accession number(s) can be found below: Harvard Dataverse: https://doi.org/10.7910/DVN/WZBHHR.

## Ethics Statement

The studies involving human participants were reviewed and approved by Texas A M University's Institutional Review Board (2019-0762D). The patients/participants provided their written informed consent to participate in this study. Written informed consent was obtained from the individual(s) for the publication of any potentially identifiable images or data included in this article.

## Author Contributions

JY designed the experiment, performed the analysis, and wrote the manuscript. CT created the experimental stimuli and wrote the manuscript. TN collected the data. All authors contributed to the article and approved the submitted version.

## Conflict of Interest

The authors declare that the research was conducted in the absence of any commercial or financial relationships that could be construed as a potential conflict of interest.
